# A mixed-methods sequential explanatory design comparison between COVID-19 infection control guidelines’ applicability and their protective value as perceived by Israeli healthcare workers, and healthcare executives’ response

**DOI:** 10.1186/s13756-020-00812-8

**Published:** 2020-09-04

**Authors:** Anat Gesser-Edelsburg, Ricky Cohen, Nour Abed Elhadi Shahbari, Rana Hijazi

**Affiliations:** 1 School of Public Health and the Health and Risk Communication Research Center, University of Haifa, 199 Aba Khoushy Ave., Mount Carmel, 3498838 Haifa, Israel; 2 Head of Health Promotion Program, School of Public Health, Founding Director of the Health and Risk Communication Research Center, University of Haifa, 199 Aba Khoushy Ave., Mount Carmel, 3498838 Haifa, Israel

**Keywords:** COVID-19, Infection control guidelines, Healthcare workers, Applicability and protective value, Perception

## Abstract

**Background:**

Healthcare workers (HCWs) are on the front line of the COVID-19 outbreak, and their constant exposure to infected patients and contaminated surfaces puts them at risk of acquiring and transmitting the infection. Therefore, they must employ protective measures. In practice, HCWs in Israel were not fully prepared for this sudden COVID-19 outbreak. This research aimed to identify and compare: (1) Israeli HCWs’ perceptions regarding the official COVID-19 guidelines’ applicability and their protective value, and (2) HCWs executives’ response to HWCs’ concern regarding personal protective equipment (PPE) shortage.

**Methods:**

A mixed-methods sequential explanatory design consists of: (1) An online survey of 242 HCWs about the application of the guidelines and PPE, and (2) Personal interviews of 15 HCWs executives regarding PPE shortage and the measures they are taking to address it.

**Results:**

A significant difference between the perceived applicability and protective value was found for most of the guidelines. Some of the guidelines were perceived as more applicable than protective (hand hygiene, signage at entrance, alcohol rub sanitizers at entrance, and mask for contact with symptomatic patients). Other were perceived as less applicable than protective (prohibited gathering of over 10 people, maintaining a distance of 2 m’, and remote services).

**Conclusions:**

HCWs need the support of the healthcare authorities not only to provide missing equipment, but also to communicate the risk to them. Conveying the information with full transparency, while addressing the uncertainty element and engaging the HCWs in evaluating the guidelines, are critical for establishing trust.

## Background

COVID-19 is a contagious viral infection, caused by newly identified virus [1]. Presentation can range from no symptoms to severe illness including pneumonia, respiratory failure, septic shock, and multi-organ failure, which may result in death [1].

There is a dispute as to how the virus is transmitted from one person to another (droplet or airborne transmission). Recently, the World Health Organization (WHO) declared that current information indicated that transmission is usually by droplets [2]. Droplet transmission occurs when a person is in close contact (within 1 m’) with someone who has respiratory symptoms (e.g., coughing or sneezing) and is therefore at risk of having his/her mucosae (mouth and nose) or conjunctiva (eyes) exposed to potentially infective respiratory droplets. However, the organization declares there is still uncertainty about airborne contagion, and concludes that “these initial findings need to be interpreted carefully” [2]. Therefore, some countries and organizations, including the US and European Centers for Disease Control and Prevention recommend airborne precautions for any situation involving the care of COVID-19 patients [3, 4].

On 30 January 2020, the WHO Director-General declared that the current outbreak constituted a public health emergency of international concern. Avoidance of exposure is the single most important measure for preventing COVID-19, so-called “social distancing.” In the case of HCWs, they must wear PPE including gloves, medical masks, goggles or a face shield, and gowns, as well as for specific procedures, respirators and aprons [1, 3].

At the end of February 2020, the WHO published recommendations for the rational use of PPE in healthcare and community settings that aims to provide information about when PPE use is most appropriate [1, 5].

In Israel the Ministry of Health (MOH) at the beginning of March published “Guidelines for dealing with COVID-19 for health professionals in healthcare settings” [6] Even though Israel was one of the first countries to understand the severity of the epidemic, and the MOH had taken drastic measures to close the country’s borders [7], the State Comptroller’s report declared that Israel is not prepared for the COVID-19 epidemic and that the MOH has no organized plan to mitigate the shortage gaps in inpatient beds, medical teams, and PPE.

Considering the critical issues regarding guidelines for dealing with COVID-19 for healthcare professionals in healthcare settings and the lack of evaluation studies regarding the implications of the formal guidelines, this article seeks to: (1) Identify the attitudes and perceptions of Israeli HCWs regarding official guidelines (their applicability, protection of HCWs, and spread prevention); (2) Present behavioral practices proposed by the front-line HCWs; and (3) present senior health executives’ response to the PPE shortage.

## Methods

### Research design

This research is based on mixed-methods sequential explanatory design [8]. The quantitative research is the main research, conducted through an online survey of 242 HCWs concerning the application of the guidelines and protective measures. The secondary qualitative research examined 15 healthcare executives’ perceptions of the HCWs’ reports of a PPE shortage, and the actions they took to address that issue using personal interviews [8].

### Sampling and data collection

An online questionnaire was designed using the Qualtrics XM software for a deliberate nonprobability sampling [9] of the population of HCWs. This method was selected because the COVID-19 crisis requires social distancing, preventing face-to-face interviews. Furthermore, HCWs are the busiest population at this time.

The survey was distributed in March 2020 to HCWs using three main social media platforms: Facebook, WhatsApp, and Instagram. The first stage was deliberate intensive sampling through posts on specific social media forums for healthcare workers, such as the forum of nurses in Israel, the forum of Arab doctors in Israel, and more. Meanwhile, the questionnaire was sent to directors of health funds and hospitals and from there was distributed to their employees on WhatsApp. The second stage was snowball sampling [10] to reach broader circles of healthcare workers.

In the qualitative research, we performed intensive sampling of health executives and senior physicians. The study was advertised through WhatsApp groups, and executives and physicians who agreed to participate in the study contacted the researchers to schedule telephone interviews. Each interview lasted 20–30 min.

### Study population

A total of 292 HCWs filled out the online questionnaire, however 50 (17%) questionnaires were not fully completed. Those questionnaires were taken out of the sample, leaving 242 HCWs (Table 1) who fully completed the questionnaire. That was done only after verifying that the main research findings did not differ when the whole set of questionnaires (292) was included in the statistical analysis.

**Table 1:** Healthcare workers socio-demographic characteristics (*N* = 242)

Characteristic	N (%)
**Sector**	
Physician	34 (14)
Nurse	109 (45)
Paramedic	61 (25)
Administration	38 (16)
**Location**	
Hospital	113 (47)
Community	129 (53)
**Gender**	
Male	71 (29)
Female	171 (71)
**Age**	
< =30	69 (29)
31–40	100 (41)
41–50	37 (15)
51–60	29 (12)
61+	7 (3)

In the qualitative research 15 health executives and senior physicians participated, including 6 (40%) hospital directors, 3 (20%) healthcare fund directors, 3 (20%) directors of medical organizations, and 3 (20%) senior physicians.

### The research process

The quantitative questionnaire consisted of questions on sociodemographic details and questions on the guidelines for HCWs for the COVID-19 crisis based on MOH guidelines [6]. The questions about the MOH guidelines included questions about the applicability of each guideline and the protection it offers the worker and the public against contagion with the COVID-19 (See Additional file 1). For each guideline, the participants were asked to rank its level of applicability, and the way they perceive its protection against their own contagion and the public’s. The ranking was based on a Likert scale from 1 (not at all) to 5 (very much). The next part of the questionnaire included open questions about the PPE and a question about other protective practices HCWs perform, which are not in the official guidelines.

After finishing collecting the quantitative data, an interesting issue emerged from the open question in the qualitative research, about a shortage of PPEs on the ground. Therefore, in the personal interviews with hospital directors and senior officials, the two following questions were included: (1) How do you perceive the issue of PPE shortage? and (2) What actions are you taking to address this issue?

### Analysis

In the first stage, we performed a statistical analysis to test the difference between the applicability of the guideline and its degree of protection/contagion prevention for each of the MOH guidelines. The comparisons between the applicability of the guidelines and the degree of protection and contagion prevention were made by a Wilcoxon Signed-Ranks test for dependent measures. Furthermore, Friedman’s test was conducted in order to check for efficacy differences among the guidelines.

In the second stage, the findings of the open questions in the questionnaire were coded into categories on the additional protective measures that do not exist in the guidelines. In the third stage the report of missing PPE was integrated with a content analysis of the personal interviews with the HCWs executives about their response to the PPE shortage.

### Reliability and validity

Before the questionnaire was distributed, a pilot study was conducted among 20 participants in order to refine the questions and prevent information bias. The questions were written in Hebrew and translated into Arabic and later re-translated to Hebrew in order to check their wording. In addition, in order to test the validity of the guidelines we presented in the questionnaire, and in order to check for sectorial differences (physicians, nurses, paramedics and administrative workers) concerning perceptions of the protection guidelines, Kruskal-Wallis tests were performed on each guideline. Three perceptions were examined for each guideline; its level of applicability, its protectiveness for the healthcare worker, and its preventive level against the public’s contagion. No significant differences were found between the 4 sectors on all these comparisons, which eliminates the need to consider this parameter in further analysis of these data (See Additional file 2).

The brief qualitative interviews with the directors were conducted in the Arabic and Hebrew languages. All of the interviews were recorded, transcribed, and translated into Hebrew. The transcription of the interviews in the Arabic language was performed by two Arabic-speaking researchers who speak both languages.

## Results

The comparison between the applicability of each guideline and its protective value was done using a Wilcoxon Signed-Ranks test for dependent measures (Table 2).

**Table 2:** Guidelines applicability average degree compared to its protective value (“protecting me” and “prevent contagion”) (*N* = 242)

Guideline	Applicable	Protect/Prevent Contagion	Test Statistic (S)^a^	***p***-value	Adjusted p^b^
Hand hygiene	4.5	4.0	4122.5	<.0001	<.0001
Gloves and gown	3.9	3.7	960.0	0.02	0.20
Signage at entrance	4.4	3.8	3190.5	<.0001	<.0001
Alcohol rub sanitizers at entrance	4.3	4.0	1593.5	<.0001	<.0001
Mask for symptomatic patients	3.8	3.9	− 446.0	0.20	0.89
Mask for contact with symptomatic patients	4.3	3.9	1923.5	<.0001	<.0001
Prohibited gathering over 10 people	3.6	3.8	− 1020.5	0.004	0.04
Maintaining a distance of 2 m’	3.0	3.7	− 3470.5	<.0001	<.0001
Questioning at entrance	3.5	3.6	− 754.5	0.08	0.58
Remote services	3.9	4.4	− 2277.0	<.0001	<.0001

^a^ Wilcoxon Signed-Ranks tests

^b^ Sidak adjustment for multiple testing

A significant difference between applicability and protective value was found for several guidelines, except for three guidelines (using gloves and gown, mask for symptomatic patients, and questioning at entrance). Some of the guidelines are perceived as more applicable than protective (hand hygiene, signage at entrance, alcohol rub sanitizers at entrance, and mask for contact with symptomatic patients). Three guidelines are perceived as less applicable than protective (prohibited gathering over 10 people, maintaining a distance of 2 m’, and remote services).

Friedman’s test was conducted in order to check for efficacy differences among the guidelines. The different guidelines were examined using an overall measure of efficacy which averages the applicability and the protective value of each guideline. Test results (Table 3) show significant differences (χ2(9) = 553.5, *P* < 0.0001) between the guidelines.

**Table 3:** A comparison of guideline applicability and protective value using an overall measure of efficacy (N = 242)

Guideline	Overall measure of efficacy^a^	Chi-Square^b^	DF	*p*-value
Hand hygiene	4.2	317.52	9	< 0.001
Remote services	4.2			
Alcohol rub sanitizers at entrance	4.2			
Signage at entrance	4.1			
Mask for contact with symptomatic patients	4.1			
Mask for symptomatic patients	3.8			
Gloves and gown	3.8			
Prohibited gathering over 10 people	3.7			
questioning at entrance	3.5			
Maintaining a distance of 2 m	3.3			

^a^ Averages the applicability and the protective value of each guideline

^b^ Friedman’s test

Additional practices that do not appear in the official guidelines against COVID-19, were suggested by the HCWs (Table 4).

**Table 4:** Additional practices suggested by the HCWs that do not appear in the official guidelines against COVID-19

Practice No.	Practice description
1	Eating healthy, vitamin C-fortified food, and drinking a lot of water
2	Rinsing nostrils with water and soap after shift
3	Disinfecting personal belongings before going home
4	Disinfecting cell phone, keyboard, mouse, and other equipment throughout the day
5	Changing clothes and disinfecting shoes when entering the house
6	Washing work clothes separately
7	Opening doors with elbow
8	Thorough cleaning of surfaces and chairs
9	Cleaning steering wheel and car door handles
10	Showering at the end of shift before going home

The HCWs also reported a shortage of PPEs. The main shortages reported (by rate of respondents) are of facemasks (19%), gowns (16%), general protective gear (10%), disinfectant (10%), gloves (7%), and surgical masks (7%).

The response of the HCWs executives to the HCWs’ concern over PPE shortages included three main themes, that are present with their selected quotes.

1. HCWs’ fears of contagion
  “As deputy director I see a tremendous shortage of equipment. It is extremely difficult to deal with the situation because my staff members are afraid of contagion during treatment.”
  “Right now, I as a physician and the medical personnel around me are very exposed to the disease. They are barely protected, and it disturbs me. When a patient comes to my room, I don't know anything about them, what their condition is, whether they were exposed or not, infected or not. Our chances of contagion are very high. There is not sufficient protective gear, there are not even temperature checks.”
  “There is a feeling that protection of the human resource is insufficient, there is neither a health nor an economic safety net.”
2. Adjusting expectations and resource allocation
  “There is tremendous anger among the staff about the equipment shortage. I try to solve it by setting an optimal protection level for use per team. The idea is to control the amounts and not let the workers go crazy...”
  “I sat with all the teams, from all the departments in the clinic, we developed a work procedure, which team members need to be protected, and then together we came up with a “portion” of PPE based on the number of staff members, the number of patients, and which needs or cases they deal with. Every morning each team gets its portion. It will prevent misunderstandings and help the team understand the situation better.”
3. Remote services.
  “We use videoconferencing in the treatment room, to reduce to a minimum the medical staff's exposure to patients and contagion.”
  “Currently the communication between the medical staff and the COVID-19 patients in the departments is remote, the communication is by intercom. The medical staff is not exposed to the confirmed patients.”

## Discussion

HCWs are on the front line of the COVID-19 outbreak, and their constant exposure to infected patients and contaminated surfaces puts them at risk for acquiring and transmitting the infection. Current global stockpiles of PPE are insufficient, driven not only by the number of COVID-19 cases but also by misinformation, panic buying and stockpiling [1, 11, 12]. Major distributors in the United States have already reported shortages of PPE [3, 13].

The findings of the present study point to the gap the HCWs perceive between the applicability of the existing guidelines and the protection of HCWs against contagion and infecting the public. These findings indicate the gap that has been documented in the literature, not only in the COVID-19 epidemic, between the official infection prevention guidelines and what happens on the ground [14].

The findings of the present study indicate that three guidelines are perceived as less applicable than protective: prohibited gathering of over 10 people, maintaining a distance of 2 m’, and remote services.

A possible interpretation to that is that the Israeli healthcare system has fewer staff positions and manpower than the Organisation for Economic Co-operation and Development (OECD) countries [15]. Israel has a tremendous overload on its healthcare system that is reflected by long lines [15–17]. The HCWs feel they cannot question the people who come in during the coronavirus crisis because they do not have enough time and there is a personnel shortage. In addition, since the hospitals and health funds are at full capacity all the time, during the COVID-19 crisis the pressure increases, and it is difficult to reduce the congregating and maintain a distance between people. Another possible explanation for the difficulty to maintain a distance is the cultural component. Israeli society has a culture of social intimacy where it is not common to keep a physical distance. Moreover, there are geographical areas in Israel with high population density where people congregate, such as the Arab and ultra-Orthodox communities. In the COVID-19 crisis in Israel certain populations have reportedly had difficulty following the guidelines, making it hard for the HCWs to maintain the specific guidelines concerning social distancing. For example, the ultra-Orthodox community in Israel found it difficult to follow the guidelines and suffered from some of the highest infection rates in the country [18, 19].

The findings also indicated that some guidelines were perceived as more applicable than protective, such as hand hygiene, and alcohol rub sanitizers at entrance. Hand hygiene (HH) is the single most effective way to reduce the spread of germs that cause respiratory disease [20]. HH after removing PPE is particularly important to remove any pathogens that might have been transferred to bare hands during the removal process [3].

One explanation for the findings of the present study that HCWs view HH as less protective of them might be the cognitive bias that exists among many of them, as it does in the general public, whereby they perceive PPE as a solution but forget to perform HH in the course of their work [21].

This interpretation is consistent with other research in the literature about the prevention of hospital-acquired infections, which indicates that despite a variety of interventions conducted for HCWs, the levels of compliance with HH still remain low at 50–60% [22–24]. during the COVID-19 crisis Reports from China indicate that suboptimal HH after contact with patients was linked to COVID-19 [25, 26]. Long exposure time to large numbers of infected patients directly increased the risk of HCW infections [11, 12].

Another guideline perceived as applicable but less protective is masks for contact with symptomatic patients. The professional literature indicates uncertainty about the effectiveness of wearing masks. Some say that wearing a mask can lead to false confidence [27]. It is possible that the dispute about the degree of protection from masks led the respondents to indicate a gap between applicability and protection. It is also possible that the uncertainty that still exists about the transmission of the coronavirus (droplet or airborne) also contributed to the gap found in regard to masks between its applicability and the degree of its protection against contagion [2].

Recent studies in the field of infection control indicate that the official guidelines focus on the temporal order of actions in their broadest sense and cannot be totally comprehensive as exigency situations arise from the dynamic nature of the work, that exist in the care continuum [28, 29].

Respectively, in a crisis such as the COVID-19 epidemic, the staff faces new situations they did not conceive of before, as the virus spreads. Therefore, in the study we asked the respondents to share with us additional practices they perform that cannot be found in existing guidelines. The HCWs raised creative practices that indicate the importance of including the staff when confronting an epidemic crisis. Guidelines that are handed down from above are insufficient in a changing reality and it is important to hear the staff and accommodate them.

This study indicates there is a PPE shortage of for HCWs in Israel like in other countries [30]. Many of the HCWs (69%) noted the PPE shortage. The HCWs executives interviewed for this research noted that the PPE shortage led to feelings of anger and frustration among the HCWs. During the crisis in Israel due to the PPE shortage for its employees, the MOH issued a statement at a press conference in mid-March, saying that HCWs do not need to wear PPE regularly but rather consider the situations in which they should do so [31].

Following the MOH statement, senior doctors from across the country came together and sent a letter to the government stating that their voices were not being heard, and that the state was abandoning them due to the severe shortage of PPE [32, 33]. The MOH statement was perceived as an excuse to cover up the inadequacy of the Israeli healthcare system, of which the PPE shortage is only one example [7].

### Limitations

The research limitation is that this is not a representative sample of all HCWs in Israel. However, the questionnaire was filled out by diverse sectors of the Jewish and Arab populations working in the Israeli healthcare system, both in the community and in hospitals. Follow-up studies might examine HCWs’ perceptions concerning the guidelines for treating COVID-19 patients: guidelines about care, their level of applicability, and suggestions from the staff can contribute important information to the healthcare system.

## Conclusions

The HCWs on the front line of this global crisis need the support of the authorities not only to provide missing equipment, but also to communicate the risks to them. Emerging infection diseases communication [34] is a critical strategy not only for conveying information to the general public, but also for HCWs [35–37]. Furthermore, including the personnel, while discussing with them the level of applicability of the guidelines and the way they perceive the risks, can help the authorities communicate with their staff effectively and adjust the guidelines to the reality on the ground. Furthermore, including the HCWs and enabling them to contribute additional solutions that can prevent infection is significant and can help the overall effort.

## Supplementary information

**Additional file 1: Table S5.** Questionnaire for healthcare workers.

**Additional file 2: Table S6.** Guideline applicability and protective value: a comparison between sectors (Kruskal-Wallis Tests) (*N*=242).

## Data Availability

Requests for more detailed information regarding the study can be addressed to the corresponding author.

## Abbreviations

HCWs: Healthcare workers
HH: Hand hygiene
MOH: Ministry of Health
PPE: Personal protective equipment
WHO: World Health Organization
