# Supplementary material for: A mixed-methods sequential explanatory design comparison between COVID-19 infection control guidelines’ applicability and their protective value as perceived by Israeli healthcare workers, and healthcare executives’ response
Source: Antimicrob Resist Infect Control. 2020 Sep 4;9:148. doi: 10.1186/s13756-020-00812-8 (PMC7472407; doi:10.1186/s13756-020-00812-8)
Supplement: Supplementary file 1 — Additional file 1: Table S5. Questionnaire for healthcare workers. [file 13756_2020_812_MOESM1_ESM.docx]

**Table S5.** **Questionnaire for healthcare workers.**

1. Following are the guidelines issued by the Israeli Ministry of Health and health funds. Please mark the answer you find most suitable (1=Not at all, 2=A little, 3=To a moderate extent, 4=A lot, 5=Very much) for each of the following statements:

| Hand hygiene, by washing hands with soap and water or a solution containing 70% alcohol, following the five-moment principles (between patients, contact with immediate surrounding, before eating and after using the bathroom) | | | | | |
| --- | --- | --- | --- | --- | --- |
| 5 | 4 | 3 | 2 | 1 | Applicable guideline |
| 5 | 4 | 3 | 2 | 1 | The guideline protects me against contagion with the coronavirus |
| 5 | 4 | 3 | 2 | 1 | The guideline prevents spread of the virus to the public |
| Use of gloves and routine body protection during contact with patients with upper respiratory symptoms and with their surroundings, including surfaces, clothing, or dirty bedding | | | | | |
| 5 | 4 | 3 | 2 | 1 | Applicable guideline |
| 5 | 4 | 3 | 2 | 1 | The guideline protects me against contagion with the coronavirus |
| 5 | 4 | 3 | 2 | 1 | The guideline prevents spread of the virus to the public |
| Post at the entrance to the clinic/treatment room or hospital a sign explaining the importance of performing hand hygiene | | | | | |
| 5 | 4 | 3 | 2 | 1 | Applicable guideline |
| 5 | 4 | 3 | 2 | 1 | The guideline protects me against contagion with the coronavirus |
| 5 | 4 | 3 | 2 | 1 | The guideline prevents spread of the virus to the public |
| Install at the entrance to the clinic/treatment room or hospital alcohol rub sanitizer | | | | | |
| 5 | 4 | 3 | 2 | 1 | Applicable guideline |
| 5 | 4 | 3 | 2 | 1 | The guideline protects me against contagion with the coronavirus |
| 5 | 4 | 3 | 2 | 1 | The guideline prevents spread of the virus to the public |
| A staff member shall give patients, whom they identify as having upper respiratory system symptoms, surgical masks, and ask them to sit as far as possible from other people | | | | | |
| 5 | 4 | 3 | 2 | 1 | Applicable guideline |
| 5 | 4 | 3 | 2 | 1 | The guideline protects me against contagion with the coronavirus |
| 5 | 4 | 3 | 2 | 1 | The guideline prevents spread of the virus to the public |
| HCWs should wear surgical masks when treating patients with upper respiratory system symptoms | | | | | |
| 5 | 4 | 3 | 2 | 1 | Applicable guideline |
| 5 | 4 | 3 | 2 | 1 | The guideline protects me against contagion with the coronavirus |
| 5 | 4 | 3 | 2 | 1 | The guideline prevents spread of the virus to the public |
| Multiple uses of the same surgical mask | | | | | |
| 5 | 4 | 3 | 2 | 1 | Applicable guideline |
| 5 | 4 | 3 | 2 | 1 | The guideline protects me against contagion with the coronavirus |
| 5 | 4 | 3 | 2 | 1 | The guideline prevents spread of the virus to the public |
| Ban on gathering of more than 10 people in the same space | | | | | |
| 5 | 4 | 3 | 2 | 1 | Applicable guideline |
| 5 | 4 | 3 | 2 | 1 | The guideline protects me against contagion with the coronavirus |
| 5 | 4 | 3 | 2 | 1 | The guideline prevents spread of the virus to the public |
| Maintaining a 2 m’ distance between people (between staff members/ the public/ staff member and patient) | | | | | |
| 5 | 4 | 3 | 2 | 1 | Applicable guideline |
| 5 | 4 | 3 | 2 | 1 | The guideline protects me against contagion with the coronavirus |
| 5 | 4 | 3 | 2 | 1 | The guideline prevents spread of the virus to the public |
| Posting a staff member at the main entrance whose job is to question the public (about return from travel abroad, spending time with a patient, and their health status) | | | | | |
| 5 | 4 | 3 | 2 | 1 | Applicable guideline |
| 5 | 4 | 3 | 2 | 1 | The guideline protects me against contagion with the coronavirus |
| 5 | 4 | 3 | 2 | 1 | The guideline prevents spread of the virus to the public |
| The public's use of remote services, such as digital services and telemedicine | | | | | |
| 5 | 4 | 3 | 2 | 1 | Applicable guideline |
| 5 | 4 | 3 | 2 | 1 | The guideline protects me against contagion with the coronavirus |
| 5 | 4 | 3 | 2 | 1 | The guideline prevents spread of the virus to the public |
| Are there staff members in your immediate surroundings who overuse protective equipment and under-practice hand hygiene? | | | | | |
| 5 | 4 | 3 | 2 | 1 |  |

1. What PPE is missing at your job?
2. Besides the guidelines known to HCWs, do you take any additional measures of your own to protect yourself and the public against contagion? Please specify.
