# Supplementary material for: A mixed-methods sequential explanatory design comparison between COVID-19 infection control guidelines’ applicability and their protective value as perceived by Israeli healthcare workers, and healthcare executives’ response
Source: Antimicrob Resist Infect Control. 2020 Sep 4;9:148. doi: 10.1186/s13756-020-00812-8 (PMC7472407; doi:10.1186/s13756-020-00812-8)
Supplement: Supplementary file 2 — Additional file 2: Table S6. Guideline applicability and protective value: a comparison between sectors (Kruskal-Wallis Tests) (N=242). [file 13756_2020_812_MOESM2_ESM.docx]

**Table S6.** **Guideline applicability and protective value: a comparison between sectors (Kruskal-Wallis Tests) (N=242).**

| **Guideline** | **Value** | **Chi-Square** | **p-value (DF=3)** | **Adjusted p** |
| --- | --- | --- | --- | --- |
| Hand hygiene | Applicable | 4.13 | 0.25 | 1.00 |
|  | Protective | 3.08 | 0.38 | 1.00 |
|  | Prevent Contagion | 1.32 | 0.72 | 1.00 |
| Gloves and gown | Applicable | 1.26 | 0.74 | 1.00 |
|  | Protective | 1.46 | 0.69 | 1.00 |
|  | Prevent Contagion | 1.34 | 0.72 | 1.00 |
| Signage at entrance | Applicable | 2.65 | 0.45 | 1.00 |
|  | Protective | 0.58 | 0.90 | 1.00 |
|  | Prevent Contagion | 1.80 | 0.61 | 1.00 |
| Alcohol rub sanitizers at entrance | Applicable | 2.20 | 0.53 | 1.00 |
|  | Protective | 0.88 | 0.83 | 1.00 |
|  | Prevent Contagion | 0.58 | 0.90 | 1.00 |
| Mask for symptomatic patients | Applicable | 1.36 | 0.71 | 1.00 |
|  | Protective | 0.06 | 1.00 | 1.00 |
|  | Prevent Contagion | 0.16 | 0.98 | 1.00 |
| Mask for contact with symptomatic patients | Applicable | 13.54 | 0.00 | 0.10 |
|  | Protective | 4.53 | 0.21 | 1.00 |
|  | Prevent Contagion | 1.530 | 0.68 | 1.00 |
| Prohibited gathering over 10 people | Applicable | 0.96 | 0.81 | 1.00 |
|  | Protective | 0.31 | 0.96 | 1.00 |
|  | Prevent Contagion | 0.53 | 0.91 | 1.00 |
| Maintaining a distance of 2m | Applicable | 4.40 | 0.22 | 1.00 |
|  | Protective | 3.26 | 0.35 | 1.00 |
|  | Prevent Contagion | 3.90 | 0.27 | 1.00 |
| Questioning at entrance | Applicable | 5.31 | 0.15 | 1.00 |
|  | Protective | 1.66 | 0.65 | 1.00 |
|  | Prevent Contagion | 3.15 | 0.37 | 1.00 |
| Remote services | Applicable | 0.62 | 0.89 | 1.00 |
|  | Protective | 6.72 | 0.08 | 0.92 |
|  | Prevent Contagion | 6.72 | 0.08 | 0.92 |
| Sidak adjustment for multiple testing. | | | | |
